# Pushing the Resolution Limit of Stimulated Emission Depletion Optical Nanoscopy

**DOI:** 10.3390/ijms25010026

**Published:** 2023-12-19

**Authors:** Sejoo Jeong, Dongbin Koh, Eunha Gwak, Chinmaya V. Srambickal, Daeha Seo, Jerker Widengren, Jong-Chan Lee

**Affiliations:** 1 Department of New Biology, DGIST, Daegu 42988, Republic of Korea; 2 School of Undergraduate Studies, DGIST, Daegu 42988, Republic of Korea; 3 Exp. Biomol. Physics, Dept. Applied Physics, KTH—Royal Institute of Technology, 106 91 Stockholm, Sweden; 4 Department of Physics and Chemistry, DGIST, Daegu 42988, Republic of Korea; 5 New Biology Research Center, DGIST, Daegu 42988, Republic of Korea

**Keywords:** super-resolution microscopy, STED, resolution, temporal resolution, fluorophore localization, adaptive illumination, fluorescence lifetime, nanobody, fluorescent protein, optical nanoscopy

## Abstract

Optical nanoscopy, also known as super-resolution optical microscopy, has provided scientists with the means to surpass the diffraction limit of light microscopy and attain new insights into nanoscopic structures and processes that were previously inaccessible. In recent decades, numerous studies have endeavored to enhance super-resolution microscopy in terms of its spatial (lateral) resolution, axial resolution, and temporal resolution. In this review, we discuss recent efforts to push the resolution limit of stimulated emission depletion (STED) optical nanoscopy across multiple dimensions, including lateral resolution, axial resolution, temporal resolution, and labeling precision. We introduce promising techniques and methodologies building on the STED concept that have emerged in the field, such as MINSTED, isotropic STED, and event-triggered STED, and evaluate their respective strengths and limitations. Moreover, we discuss trade-off relationships that exist in far-field optical microscopy and how they come about in STED optical nanoscopy. By examining the latest developments addressing these aspects, we aim to provide an updated overview of the current state of STED nanoscopy and its potential for future research.

## 1. Introduction

**Figure 1:**
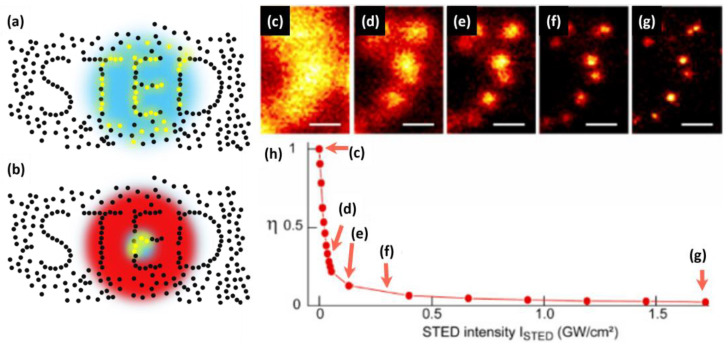
The principle of the STED optical nanoscopy and the resolution improvement as a function of increasing the STED power. (**a**) Excitation point spread function (PSF) of a conventional confocal microscope. Blue is the excitation PSF; yellow dots are fluorophores that are in fluorescent state. (**b**) Effective PSF of STED nanoscopy achieved by adopting a donut-shaped STED beam (shown in red). (**c**–**g**) STED images of 24 nm fluorescent beads on a cover slip recorded with different STED intensities. (**h**) STED depletion measured on the same sample. The intensity settings for the measurements (**c**–**g**) are marked by red arrows. Scale bar in (**c**–**g**) 200 nm. (**c**–**h**) Reproduced from Ref. [10] with permission from the Optica Publishing Group.

Light microscopy, or optical microscopy, has been a cornerstone of life science research for more than a century, but its resolution has long been limited by Abbe’s diffraction limit [1]. This limitation has constrained the observation of structures and processes on a smaller scale, impeding our understanding of the cellular world. However, over the past couple of decades, several techniques, which are called optical nanoscopy or super-resolution optical microscopy, have circumvented this limit and achieved resolution far beyond the diffraction limit [2,3,4,5,6,7]. Single-molecule localization microscopy (SMLM), such as photoactivated localization microscopy (PALM) [2] or stochastic optical reconstruction microscopy (STORM) [3], circumvents Abbe’s diffraction limit utilizing the photoactivation or photo-switching of fluorescent molecules to isolate and precisely localize individual molecules in sequential imaging frames. Structured illumination microscopy (SIM) uses spatially structured illumination patterns to generate moiré fringes, which enable the transmission and acquisition of somewhat higher spatial frequency signals [4]. Stimulated emission depletion (STED) optical nanoscopy, on the other hand, achieves super resolution by using a focused excitation beam and a donut-shaped depletion beam to selectively deplete fluorescence from the periphery of the focal spot (Figure 1a,b) [5,6]. This “resolution revolution” via optical nanoscopy has significantly contributed to the field of life sciences by providing unprecedented insights into cellular structures and dynamics beyond the limits of conventional microscopy [8]. This groundbreaking contribution was recognized by the Nobel Prize in Chemistry, which was awarded to E. Betzig, S. Hell, and W.E. Moerner in 2014.

STED optical nanoscopy has certain advantages over other super-resolution optical microscopy methods. STED nanoscopy has a better resolution compared to SIM, which harbors limited resolution improvement. The resolution of (linear) SIM is two times better than that of conventional light microscopy, whereas STED can achieve an arbitrarily good resolution [4]. Moreover, unlike SIM, STED microscopy does not require the post processing of the obtained data. Compared to SMLM, STED has a better temporal resolution in general. Due to the stochastic nature of fluorophore photoactivation, SMLM requires the acquisition of tens of thousands of images to reconstruct one super-resolved image, resulting in low temporal resolution [5,6]. In contrast, the temporal resolution of STED nanoscopy can be similar to that of confocal microscopy. Consequently, SMLM is not well suited for observing rapidly changing biological processes or samples with a high molecular density. STED holds other advantages over SMLM, as well. Firstly, there is no need for specialized buffers. Secondly, it does not require the fluorophore to exhibit specific blinking capabilities, including low duty cycles of its emissive state. Thirdly, STED does not involve post-processing steps. Moreover, it provides a better imaging depth, a notable advantage considering that SMLM often relies on total internal reflection fluorescence (TIRF) microscopy.

The spatial resolution of STED optical nanoscopy can, in principle, be arbitrarily improved simply through increasing the power of the STED beam (Figure 1c–h) [9,10]. For instance, the nanometric resolution of STED nanoscopy was experimentally demonstrated in nitrogen-vacancy centers present in optically stable nanodiamonds [11]. In practical imaging tasks, including the imaging of biological samples, however, increasing the STED beam power to improve the resolution would be often impractical. Increasing the power of the STED beam exacerbates photobleaching and photodamage in samples, causes significant background noise problems, and results in diminishment in temporal resolution as a trade-off [12,13,14,15]. Hence, it is valid to recognize that the achievable resolution in practical applications of STED nanoscopy exhibits limitations. Recent technical developments have been explored, to push the practical, usable resolution limit of STED nanoscopy by addressing these trade-offs. For instance, significant endeavors have been undertaken to mitigate the challenge of background noise in STED optical nanoscopy by, for example, using recording and subtraction of images with direct STED excitation [14], time gating [16], additional Gaussian STED pulse with time-correlated detection [17], polarization switching [13], and differential stimulated emission depletion [18]. Another pertinent aspect to highlight is that, although the temporal resolution of STED nanoscopy aligns with that of confocal microscopy, its practical temporal resolution is effectively lower. This reduction stems from the necessity to scan the sample with a greater number of pixels, a requisite attributed to the improved spatial resolution intrinsic to STED nanoscopy and the Nyquist sampling theorem. Therefore, improving the temporal resolution of STED nanoscopy while maintaining a high spatial resolution and large field of view is a non-trivial task [19]. It is crucial to pay close attention to such trade-off relations when developing methodologies aimed at improving the practical resolution in STED nanoscopy.

In this review, we explore recent advancements in the practical resolution of STED optical nanoscopy across multiple dimensions. Firstly, we introduce recently developed methods that enhance the spatial resolution, mainly in the lateral direction, utilizing fluorophore localization principles [20,21,22], adaptive STED illumination [23,24,25,26], and fluorescence lifetime detection [16,27,28,29,30,31,32]. Subsequently, we discuss the efforts to achieve super-resolution in the axial direction by adopting an additional STED pattern [10,13,33] or an additional counter-propagating STED beam to form an interference pattern [34,35]. Thirdly, we present recent endeavors to improve temporal resolution by parallelizing STED donut beams [36,37] or by conditionally triggering STED observations [19]. Finally, we delve into recent developments in labeling strategies, which bear a close relationship to spatial resolution [38,39,40,41,42,43,44,45,46,47,48,49,50,51,52,53,54,55]. By examining the latest progress in each of these domains, we aim to provide an up-to-date and comprehensive overview of the present state of STED nanoscopy, as well as its potential in future research.

## 2. Lateral Resolution Improvement

The resolution of STED optical nanoscopy can be calculated from the normalized excitation probability using the confocal excitation beam and the resulting fluorescence probability that is defined by an overlaid donut-shaped STED depletion beam [9]. The normalized excitation probability from the confocal excitation beam can be written as,

(1) $$h_{exc}\left(r\right)=C\cos^{2}\left(\pi rn\sin\alpha /\lambda_{exc}\right),$$

where C is a constant, n is the refractive index, nsin⁡α is the numerical aperture, and $\lambda_{exc}$ is the excitation wavelength. The fluorescence probability $p\left(r\right)$, as imposed by the STED beam, can be written as,

(2) $$p\left(r\right)=e^{-\sigma \tau I_{STED}(r)},$$

where σ is a cross-section for stimulated emission, τ is a STED pulse duration, and $I_{STED}\left(r\right)=I_{STED}^{max}\sin^{2}\left(\pi rn\sin\alpha /\lambda_{STED}\right)$ is the intensity profile of the STED beam, $I_{STED}^{max}$ is the maximum intensity of the STED beam, and $\lambda_{STED}$ is the STED wavelength. Then, the effective point spread function (PSF) of STED nanoscopy can be written as,

(3) $$h\left(r\right)=h_{exc}\left(r\right)\cdot p\left(r\right)=C\cos^{2}(\pi rn\sin\alpha /\lambda_{exc})\cdot e^{-(\sigma \tau /h\nu )I_{STED}(r)}.$$

This can then be re-expressed as,

(4) $$h\left(r\right)=C\cos^{2}(\pi rn\sin\alpha /\lambda_{exc})\cdot \exp\left[-\varsigma \sin^{2}\left(\pi rn\sin\alpha /\lambda_{STED}\right)\right],$$

where $I_{sat}=h\nu /\sigma \tau$, and $\varsigma =I_{STED}^{max}/I_{sat}$. We can further simplify the equation by assuming $\lambda_{exc}\approx \lambda_{STED}\equiv \lambda$ and approximating it by the Taylor series to the second order at r=0, which can be calculated as:

(5) $$h\left(r\right)=C-\frac{C\pi^{2}n^{2}\sin^{2}\alpha }{\lambda^{2}}\left(1+\varsigma \right)r^{2}.$$

The full width at half maximum (FWHM) of the STED nanoscopy ($\Delta r_{STED}$) can then be derived as:

(6) $$∴\Delta r_{STED}=\frac{\sqrt{2}}{\pi }\frac{\lambda }{n\sin\alpha }\frac{1}{\sqrt{1+\varsigma }}\approx 0.45\frac{\lambda }{n\sin\alpha }\frac{1}{\sqrt{1+\varsigma }}.$$

The power of the STED beam, which is encoded in the term ς, is a crucial parameter that affects resolution in STED nanoscopy [56,57]. Higher STED power improves spatial resolution by reducing the area where molecules remain “on” (Figure 1c–h). However, high-intensity light can cause photobleaching, phototoxicity, and background noise, limiting the practical resolution improvement [45,57,58,59]. In addition, the optimal STED power depends on various factors, such as the fluorophore used [60], sample conditions [43,61], and imaging parameters [62,63]. Therefore, careful optimization of imaging parameters and appropriate labeling strategies are required to achieve high-resolution imaging with minimal sample damage and photobleaching. There have been novel methods recently developed to improve the practical spatial resolution of STED nanoscopy by taking advantage of the principle of SMLM, adaptive illumination, and fluorescence lifetime detection.

### 2.1. Fluorophore Localization

STED nanoscopy and SMLM, two distinct super-resolution microscopy techniques, were originally developed independently. SMLM demonstrates a resolution improvement by fitting single-molecule fluorescence images with Gaussian functions. Therefore, the resolution improvement is contingent upon the number of detected fluorescent photons per molecule: $\sigma_{SMLM}\approx s/\sqrt{N}$, where $\sigma_{SMLM}$ is the standard error in the fitted position using SMLM, s is the standard deviation of the PSF of the emitter, and N is the number of photons captured from the fluorescent molecule [2,3]. However, recent research has led to efforts to merge the principles of these techniques in order to achieve nanometer or even sub-nanometer resolution in imaging and molecular tracking. The integration of STED nanoscopy and SMLM has demonstrated the capability to enhance the resolution threshold to the nanometer scale, allowing researchers to observe biological structures and processes with an unprecedented degree of precision and detail.

#### 2.1.1. LocSTED

**Figure 2:**
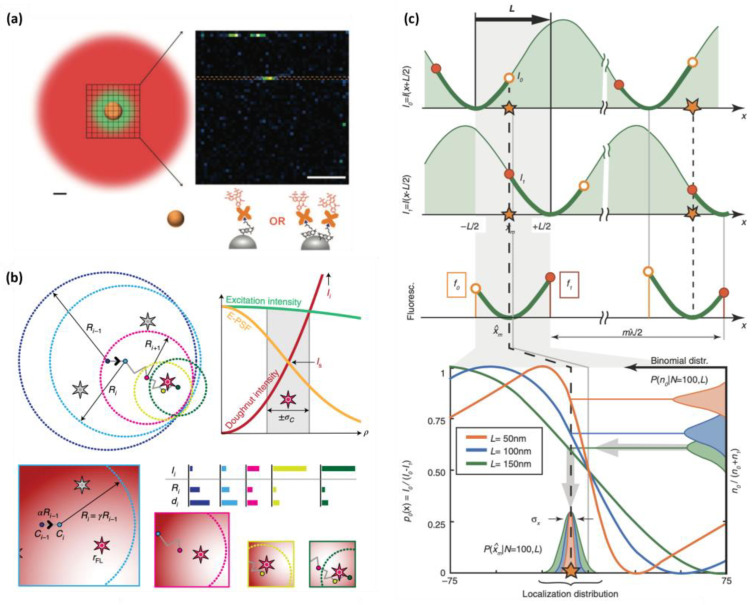
The working principle of (**a**) LocSTED, (**b**) MINSTED, and (**c**) MINFLUX. (**a**) The sub-diffraction area near the center of the STED donut is scanned for single-molecule blinking events, which are fitted with Gaussian functions to localize the centers for further resolution improvement. Reproduced from Ref. [20] with permission. Creative Commons Attribution 4.0 International License. (**b**) The STED donut beam is steered around the activated fluorophore, of which the initial position is unknown, to localize the photoactivatable fluorophore position. Subsequent donut beam steering is carried out until the donut beam center is at the fluorophore position, where the fluorescence intensity is maximum. Reproduced from Ref. [21] with permission. Creative Commons Attribution 4.0 International License. (**c**) A donut-shaped excitation beam is steered around the activated fluorophore, until the donut beam center is at the fluorophore position where the fluorescence intensity is minimum. The illustration is expressed in one dimension (x), while the principle is the same in two dimensions. Reproduced from Ref. [66] with permission from the AAAS.

The localization STED (LocSTED) technique integrates the localization principle of SMLM directly into STED nanoscopy [20]. Unlike traditional STED techniques that rely solely on depletion beams to minimize the effective PSF, LocSTED utilizes single-molecule localization to further enhance the resolution. The fluorescence PSF is initially reduced to that of STED nanoscopy via selective depletion at the periphery of the excitation focal spot. Subsequently, the sub-diffraction area near the center of the STED donut is scanned to locate single molecules which are in the photoblinking condition. These imaged molecules situated at the center of the STED donut are then fitted with Gaussian functions and localized, similarly to the SMLM principle (Figure 2a). The combination of STED and SMLM principles in LocSTED offers an improved resolution compared to that of STED alone. LocSTED thus offers a more relaxed requirement for STED beams to achieve high resolution and can be implemented using simple and cost-effective CW STED lasers while still maintaining the desired resolution [20]. However, LocSTED relies on the reversible blinking of fluorophores, which require specialized imaging buffers [61,64,65] and careful control of photobleaching to achieve optimal results. The limited temporal resolution inherent in SMLM is a shared disadvantage that also affects the performance of LocSTED microscopy.

#### 2.1.2. MINSTED

MINSTED nanoscopy uses a switching mechanism from SMLM to activate and deactivate single fluorophores, allowing only one molecule to emit fluorescence within the donut-shaped STED beam at any given time [21]. The activated fluorophore’s initial position is unknown, but the central minimum of the doughnut serves as a reference coordinate which can be steered around and used for localization with nanometric precision (Figure 2b). In terms of the application of centroid measurements or fluorophore localization, MINSTED shares similarities to MINFLUX nanoscopy [66,67,68,69]. In MINFLUX nanoscopy, typically, a donut excitation beam is steered around the fluorophore and localizes it by finding the donut minima rather than finding the local maxima as in SMLM (Figure 2c). On the other hand, MINSTED narrows down the effective PSF below the diffraction limit through the principle of STED nanoscopy, making the resolution better and particle finding more efficient compared to MINFLUX. The utilization of a strong STED depletion beam for precise localization in MINSTED brings forth an inevitable consequence of anti-Stokes background noise, which is generated via direct excitation from the STED beam, and sample photodamage [13,15]. These problems were mitigated via the adoption of a blue-shifted STED beam for MINSTED [22]. The shorter-wavelength STED beam is closer to the emission maximum of the fluorophore, and thus has a larger stimulated emission cross-section, which allows for a lower STED beam power to be used [62]. A recent MINSTED demonstration reports localization and imaging with sub-nanometer resolution using a blue-shifted STED beam [22].

### 2.2. Adaptive Illumination

The use of a high-power STED beam to improve the spatial resolution in STED optical nanoscopy can introduce various problems, such as photobleaching, phototoxicity, and background noise [45,58]. To address this problem, researchers have developed a general category of strategies called adaptive illumination. These strategies involve actively manipulating illumination or adopting an additional illumination beam to mitigate the negative effects of high-power STED beams. The adaptive illumination approaches such as RESCue, MINFIELD, DyMin, and protected STED have improved the resolution and reduced photodamage and photobleaching in STED nanoscopy, each method with its specific advantages and limitations.

#### 2.2.1. RESCue

The RESCue (reduction of state transition cycles) technique has been proposed as a method to enhance the resolution and reduce photobleaching in STED nanoscopy by actively manipulating the illumination [23]. In conventional STED nanoscopy, the high-power STED beam is illuminated continuously, regardless of whether there are features to be discriminated. However, in RESCue STED nanoscopy, the STED donut beam is intelligently switched on only when the adjoining features need to be separated, while, otherwise, the STED donut beam is intermittently turned on with a low duty cycle. This approach minimizes the time that the STED beam is on during sub-diffraction far-field imaging, which can reduce the overall light dose and photobleaching, even at high peak STED power. Consequently, RESCue STED nanoscopy yields a better practical resolution and signal-to-noise ratio than conventional STED nanoscopy.

#### 2.2.2. MINFIELD

MINFIELD STED nanoscopy is an adaptive illumination strategy that leverages a reduced phototoxicity at the center of the STED donut beam to achieve high-resolution imaging of nanoscopic features in samples [24]. Similar to the RESCue technique [23], MINFIELD avoids illuminating the entire field of view with the high-power STED beam. Instead, it selectively turns on the STED donut beam in close proximity to the features of interest, effectively causing them to fall within the low-power center of the donut beam. Utilizing the MINFIELD technique, it is possible to increase the local power of the STED beam, thereby further improving the resolution beyond what is achievable with conventional STED nanoscopy, while minimizing photobleaching and photodamage. However, MINFIELD-STED nanoscopy does have some limitations. In particular, the targeted feature must be smaller than the diffraction-limited central hole in the STED donut beam. Additionally, the application of the MINFIELD technique requires prior knowledge of the fluorophore distribution, necessitating the performance of a confocal scan beforehand.

#### 2.2.3. DyMin

**Figure 3:**
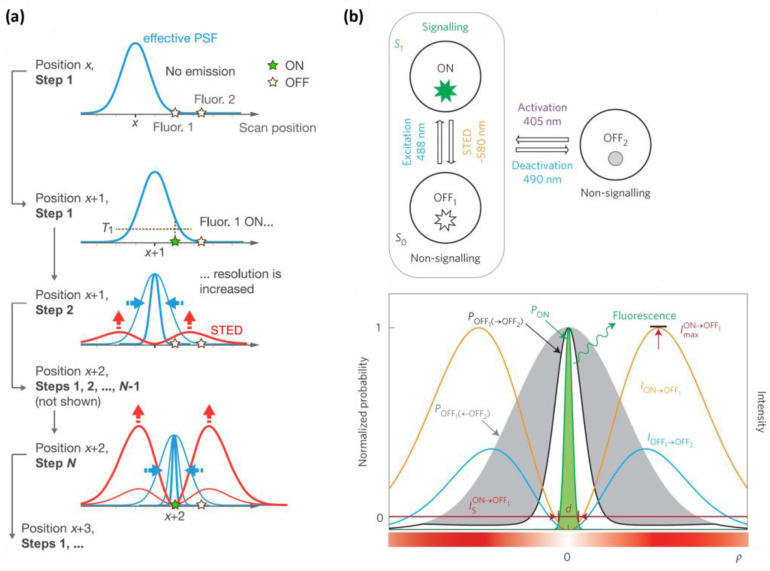
The working principle of (**a**) DyMin and (**b**) protected STED. (**a**) While scanning, the STED intensity is dynamically modulated to use high STED power only when it is required. It effectively reduces the overall STED dose and allows the use of higher STED power at the same level of phototoxicity for an enhanced resolution. Reproduced from Ref. [25] with permission from the PNAS. (**b**) Using the principle of RESOLFT, the fluorophores which are at the periphery can be turned off before STED imaging, protecting them from undergoing multiple absorption–emission cycles. Reproduced from Ref. [26] with permission from Springer Nature.

The DyMin (dynamic intensity minimum) technique is a novel adaptive illumination strategy that enhances STED resolution by selectively reducing areas where maximum STED power is applied, resulting in reduced photodamage and photobleaching [25]. Unlike the MINFIELD method, DyMin does not require prior knowledge, as it dynamically adjusts the STED power during sample scanning. The technique involves a multi-step probing approach that tracks the position and shape of the excitation beam, enabling an optimal depletion pattern for each location (Figure 3a). The full resolution power is applied only when successive increases in STED power confirm the presence of sample structures which require a higher STED power to be resolved. If no structure is detected, STED illumination is shut off immediately, reducing overall photobleaching by orders of magnitude. DyMin can, thus, achieve a resolution of 20–30 nm with reduced photodamage and photobleaching. However, the technique is complex and requires a sophisticated control algorithm, and its sensitivity to the properties of the sample may pose challenges in some applications.

#### 2.2.4. Protected STED

Protected STED is an adaptive illumination technique that employs a protection beam in conjunction with a depletion beam to minimize photodamage and photobleaching, while achieving a high resolution [26]. After activating the photo-switchable fluorophore, the donut-shaped protection beam deactivates the fluorophores, preventing them from photodamaging or photobleaching as in RESOLFT (reversible saturable optical fluorescence transitions) nanoscopy [60]. Subsequently, the high-power STED depletion beam de-excites the protected fluorophores, resulting in a sharper image (Figure 3b). Therefore, the practical resolution can be enhanced using a higher-power STED beam in protected STED. One advantage of protected STED is the ability to employ fluorescent probes with low photostability. However, the technique entails some limitations, such as requiring greater precision in beam alignment and increasing complexity. Moreover, it suffers from compromised time resolution.

#### 2.2.5. Differential STED

Differential STED (diffSTED) is a technique that can also be broadly categorized as an adaptive illumination technique [18]. DiffSTED nanoscopy achieves simultaneous enhancements in lateral and axial resolution, as well as in the signal-to-background ratio (SBR), compared to the conventional STED optical nanoscopy [18]. In diffSTED nanoscopy, signals obtained with two different STED beam intensities are used to extrapolate a final image with low background noise and intrinsically higher spatial resolution. Theoretically, the intrinsic resolution improvement can be calculated as:

(7) $$∆r_{\mathrm{diffSTED}}=0.45\frac{\lambda }{n\sin\alpha }\frac{1}{\sqrt{1+\left(\frac{1-\gamma \eta }{1-\gamma }\right)\varsigma }},$$

where *γ* is the subtraction factor, and *η* is the power ratio between the two intensities of the STED beams.

Note that other background subtraction methods, for example, polarization-switching STED (psSTED) [13], achieve improvements in STED resolution in practice by mitigating the background noise in a high STED intensity regime. DiffSTED nanoscopy, on the other hand, exhibits intrinsic resolution improvement as well as the practical resolution enhancement via higher SBR.

### 2.3. Fluorescence Lifetime Detection

Fluorescence lifetime is an inherent characteristic of a fluorophore, and can be influenced by various extrinsic factors, such as temperature, pH, polarity, and fluorescence quenchers. Not only does STED nanoscopy reduce fluorescence intensity, but the STED beam thereby also causes a reduction in the fluorescence lifetime of fluorophores located at the periphery of the donut-shaped beam. This phenomenon suggests that fluorescence lifetime detection could be leveraged to attain further contrast in STED imaging.

#### 2.3.1. g-STED

**Figure 4:**
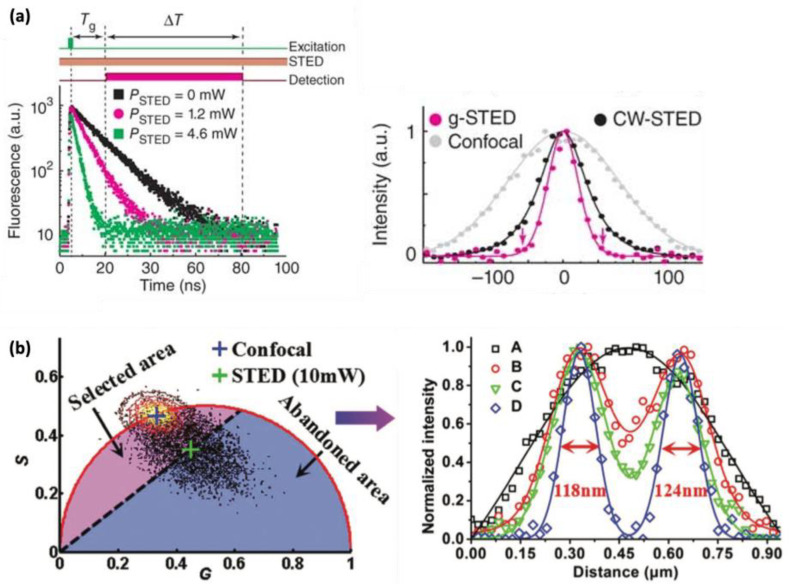
(**a**) The working principle of g-STED (**left**) and the resolution improvement (**right**). STED not only depletes the fluorescence intensity, it also shortens the fluorescence lifetime. Through time gating with some interval T_g_ after excitation, the photons under the STED beam can be better filtered out; therefore, the resolution is effectively improved. (**b**) The working principle of STED-FLIM with a phasor plot (**left**) and the resolution improvement (**right**). A: confocal, B: STED, C: g-STED, D: STED-FLIM with a phasor plot. Through adopting a temporal phasor plot, the lifetime-based filtering can be more effective. (**a**) Reproduced from Ref. [16] with permission from Springer Nature. (**b**) Reproduced from Ref. [31] with permission from The Royal Society of Chemistry.

The gated stimulated emission depletion (g-STED) approach is a time-gated methodology that enhances the on–off contrast in fluorescence, consequently improving the resolution of STED imaging [16,27,28]. As the STED donut reduces the fluorescence lifetime of fluorophores at the periphery, enhanced contrast and resolution are achieved by selectively detecting longer lifetime photons through gating the photon arrival time (Figure 4a, left). After time gating, the fluorescent molecules situated in the center of the STED beam remain detected, while those located at the periphery are further excluded and detected less. As a result, g-STED excludes low-spatial-frequency signals from the image, leading to an improvement in resolution (Figure 4a, right). The g-STED approach is particularly useful when a continuous-wave STED beam is employed. The gain in resolution with g-STED comes with the downside of a reduced overall fluorescence signal, making it unsuitable for samples with low brightness. In principle, g-STED has an advantage over other methods that use fluorescence lifetime detection in that it does not require time-correlated single-photon counting (TCSPC), as it can be replaced with a simpler time gating circuitry.

#### 2.3.2. SPLIT STED

The SPLIT (separation of photons by lifetime tuning) method has been developed to enhance the resolution of STED nanoscopy by utilizing fluorescence lifetime measurement and phasor analysis [70] to differentiate between different components with varying lifetimes [29,30]. In this approach, the total number of photons detected at each pixel includes contributions from various components, but the vector associated with the intensity decay at each pixel can be expressed as a linear combination of the vectors associated with each component that exhibits a distinct lifetime. Consequently, the different dynamic components associated with different spatial positions within the STED donut can be effectively separated, leading to a further improvement in resolution. Unlike the g-STED approach, which indiscriminately loses all signal through time gating, even when fluorescent molecules are located at the center of the donut beam, SPLIT STED can recover all photons emanating from fluorescent molecules at the center of the donut beam by selectively detecting photons with an unaffected lifetime. SPLIT STED also effectively suppresses background noise by separating the different dynamic components using fluorescence lifetime detection. However, this approach requires time-correlated single-photon counting (TCSPC) to operate, which adds complexity to the setup.

#### 2.3.3. STED-FLIM with Phasor Plot

STED in combination with FLIM (fluorescence lifetime imaging microscopy) and phasor plot analysis has emerged as a promising tool for achieving spatially super-resolved fluorescence lifetime information, which can be further utilized for enhancing spatial resolution [31,32]. In comparison with the g-STED technique, the STED-FLIM approach with phasor plot analysis is capable of better discriminating high-spatial-frequency components from low-spatial-frequency components, based on the full lifetime scatter data in phasor plot graphs. In the STED-FLIM with a phasor plot approach, the phasor plot is divided into two areas, with one area containing useful photons emerging from the center of the donut beam, while the other area typically contains unwanted photons from the periphery of the donut beam (Figure 4b, left). By selecting the area with useful photons and discarding the other area, the STED-FLIM technique with phasor plot analysis demonstrates a superior resolution compared to conventional STED and g-STED (Figure 4b, right).

The combination of fluorescence lifetime detection with STED nanoscopy, as observed in techniques such as g-STED, SPLIT STED, and STED FLIM with phasor plot, has resulted in an enhancement in resolution capabilities. However, these methods require the implementation of TCSPC or time-gating circuitry, which increases the complexity of the experimental setup and reduces the temporal resolution or signal intensity to a considerable extent. It is also worth mentioning that fluorescence lifetime correlation spectroscopy (FLCS) [71,72] has been applied to STED nanoscopy in combination with metal-induced energy transfer (MIET) to further increase the axial sectioning capability [73]. The integration of fluorescence lifetime detection with STED nanoscopy, despite the associated increase in complexity and reduction in signal intensity, offers great potential for various applications, provided the rich information from fluorescence lifetime that can be effectively utilized.

## 3. Axial Resolution Improvement

In the field of super-resolution optical microscopy, achieving high-quality imaging of complex 3D structures requires equal attention to both the axial and lateral resolution. However, according to Abbe’s diffraction limit, the axial resolution is typically inferior to the lateral resolution. Recent advancements in applying the principle of STED to enhance axial resolution have enabled significant improvements in this regard. By selectively suppressing fluorescence emissions originating outside the focal plane, the axial resolution can now approach the lateral resolution, providing high-quality imaging capabilities for complex 3D structures.

### 3.1. 3D-STED

**Figure 5:**
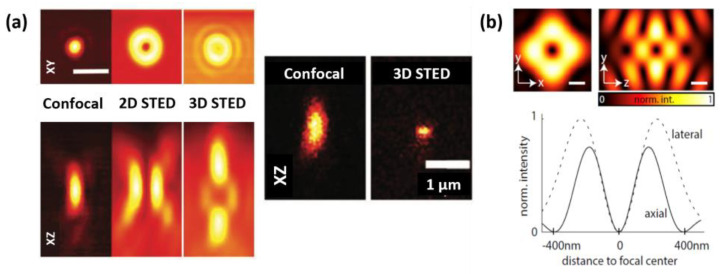
The working principle of (**a**) 3D-STED and (**b**) isoSTED. (**a**) 3D STED profile, which is obtained by the 0-pi phase plate before the objective lens, can be adopted to enhance axial resolution in STED nanoscopy. Reproduced from Ref. [10] with permission. Creative Commons Attribution 4.0 International License. (**b**) By illuminating the STED donut beam via two opposing objective lenses, the donut beam can be made to achieve isotropic resolution in both the lateral and axial directions. Reproduced from Ref. [34] with permission from Optica Publishing Group.

3D-STED microscopy is a specialized form of STED microscopy that facilitates imaging in three dimensions [10,13,33]. The conventional donut-shaped STED beam sharpens the effective PSF in the lateral dimension but leaves it unchanged in the axial dimension, resulting in an elongated effective PSF in the axial direction. In contrast, 3D-STED microscopy employs an additional STED pattern that fills in above and below the conventional STED donut beam, resulting in the depletion of fluorescence in all three dimensions (Figure 5a). This enables 3D-STED microscopy to provide a high resolution in all three dimensions, which is particularly useful for imaging complex 3D structures. The advantage of 3D-STED microscopy is the relatively simple implementation of axial resolution improvement. Notwithstanding its high axial resolution, 3D-STED microscopy suffers from the undesired depletion of signals due to elevated residual STED illumination at the center of the combined STED beam, as well as the susceptibility of the elongated donut-shaped depletion beam to optical aberrations, which thereby limits its effectiveness in thick samples.

### 3.2. Isotropic STED

Isotropic STED (IsoSTED) nanoscopy is a super-resolution technique that addresses the lack of axial resolution improvement in conventional STED microscopy by utilizing a 4Pi fluorescence collection angle arrangement, in which the sample is illuminated coherently through two opposing lenses. The symmetrical depletion spot is achieved by having two opposing STED beams with a 90-degree phase difference meet at the focus of two opposing lenses (Figure 5b). This technique enables an isotopically improved resolution in all three dimensions simultaneously, making it suitable for imaging complex biological structures in 3D [34,35]. However, the symmetrical depletion spot is achieved using two opposing STED beams, of which the coherent superposition can often be challenging to control, and the instrumentation requires the alignment of an additional beam path to have two opposing beams. Furthermore, the two opposing lenses pose a limitation to the sample volume that can be mounted and imaged, making it challenging to image large samples.

## 4. Temporal Resolution Improvement

While STED nanoscopy offers a high spatial resolution, its temporal resolution is often compromised as a trade-off. However, temporal resolution is also a crucial factor in optical microscopy, and obtaining it in STED nanoscopy may be essential for investigating nanoscale subcellular dynamics in live cells in real time. For live cell imaging, several strategies have been applied to improve the temporal resolution of STED nanoscopy. Temporal resolution, in terms of imaging throughput, is also of high relevance in imaging applications where many cells (also including fixed ones) need to be imaged at a high resolution in a limited time. In this context, using STED imaging of fixed cells, the spatial distribution patterns of specific proteins have been found to reflect mechanisms and progression, e.g., in bacterial infection [74,75,76,77] and cancer [78,79,80,81]. As a step towards diagnostic applications, this has also prompted developments of automated, faster STED imaging procedures with higher throughput [82].

### 4.1. Labeling Methods for Fast, Live Cell STED Nanoscopy

The use of stimulated emission depletion (STED) nanoscopy for high-speed live cell imaging required advancements in both optical and technical development, as well as the improvement of fluorescent probes capable of labeling biomolecules in living cells. Genetically encoded fluorescent proteins and tags have been crucial for fluorescence microscopy in general, as they enable the easy fluorescent labeling of biomolecules within living cells. However, fluorescent proteins are known to have lower brightness and photostability compared to organic dyes, which is a crucial factor in STED nanoscopy when a strong STED beam is employed. Despite this, there are numerous reports on STED imaging of live cells, or even in vivo, based on fluorescent proteins [83,84,85,86,87]. Along the quest to devise new labeling strategies that enable fast, live cell STED nanoscopy, substantial efforts have been undertaken to develop fluorescent proteins that either emit in the far-red-to-near-infrared (NIR) wavelength range, offer inherently lower phototoxicity, or exhibit enhanced fluorescence brightness, thereby rendering them highly suitable for STED nanoscopy [41,42,43,44,45,46,47,48,49,50]. It should be noted that the use of existing organic fluorophores is also a viable option for live cell STED nanoscopy through employing genetically encoded tags and self-labeling strategies. This approach has been investigated in several studies and has demonstrated the potential to expand the range of probes available for STED nanoscopy [51,52,53].

### 4.2. Parallelized Illumination

**Figure 6:**
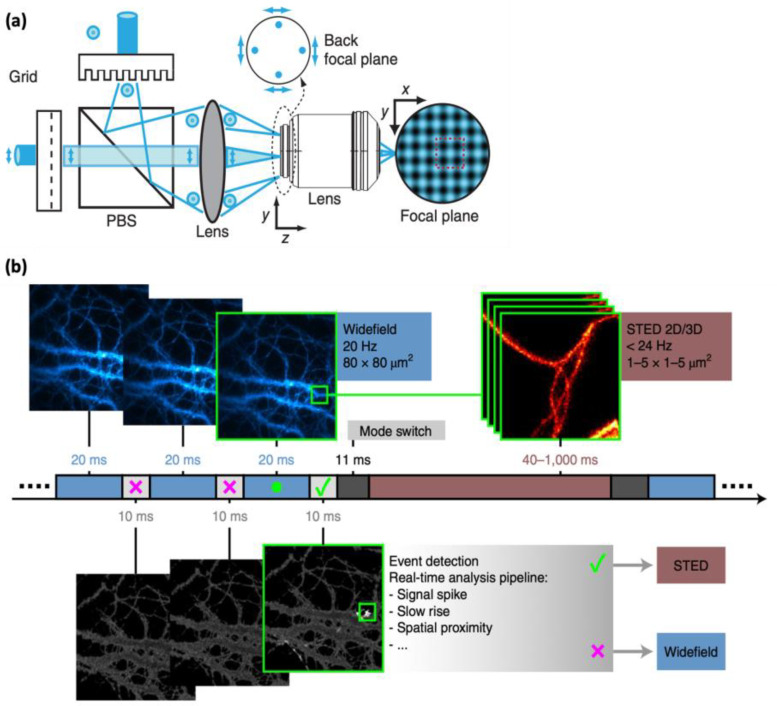
The working principle of (**a**) parallel RESOLFT and (**b**) event-triggered STED. (**a**) Two beams that pass through two orthogonal diffraction gratings can be combined to generate a parallel donut beam that can be used for parallel RESOLFT or STED. Reproduced from Ref. [88] with permission from Springer Nature. (**b**) To reduce the total dose of STED power, continuous sample monitoring, which does not require super resolution, is performed with widefield illumination, while, at the emergence of an event, the STED imaging is performed. Reproduced from Ref. [19] with permission. Creative Commons Attribution 4.0 International License.

In the last decade, the development of parallelized illumination has emerged as a promising strategy to enhance temporal resolution in STED and RESOLFT nanoscopy [36,37,88,89,90]. This strategy involves the division of the excitation and STED beams into multiple beams and scanning the sample with multiple beams simultaneously, enabling concurrent imaging of multiple points on the sample (Figure 6a). As a result, parallelized illumination has the potential to significantly increase the image acquisition speed and improve the temporal resolution. This technique has been successfully implemented in STED nanoscopy, facilitating high-temporal-resolution imaging of live cells at the speed of a few frames per second [36,37]. Despite its advantages, parallelized illumination STED nanoscopy presents certain limitations, such as the requirement for complex optics and multiple lasers, which can be expensive and challenging to align and maintain. The division of light energy into multiple donuts in parallelized STED necessitates the use of a very-high-power laser to properly deplete the fluorophores. Using a RESOLFT instead of a STED scheme will allow much lower excitation intensities to be applied, with lower phototoxicity, onto the cells, but is also inherently slower [89,90]. Also, for both STED and RESOLFT, the overlap of beams during parallel scanning may reduce the resolution at the edges of the image.

### 4.3. Event-Triggered STED (etSTED)

The development of event-triggered STED (etSTED) microscopy has addressed the fundamental trade-off between temporal and spatial resolution in biological imaging, by enabling the selective and high-resolution imaging of small areas that are identified as biologically relevant [19]. etSTED combines widefield imaging for constant monitoring of relevant events with STED nanoscopy for imaging a targeted region of interest with high spatial resolution. Upon detection of subcellular events such as local protein recruitment or vesicle trafficking, etSTED automatically triggers rapid and high-resolution 2D and 3D STED image acquisition at the site of interest (Figure 6b). Compared to traditional STED imaging, etSTED offers several advantages, including improved temporal resolution, minimized photodamage and photobleaching, and the ability to investigate various triggering events and fine subcellular structures. However, there are certain limitations to etSTED, such as the detection speed of events of interest, whereby events can be missed if they occur more rapidly than the time required to switch between the two imaging modalities. Moreover, the setup of etSTED is more complex, requiring both widefield and STED imaging capabilities and the ability to rapidly switch between the two modalities. Furthermore, etSTED is currently limited to imaging endocytosis or exocytosis, as event-specific analysis pipelines need to be developed for other specific applications. Nonetheless, the principle of etSTED is valid and has potential for further development and applications.

## 5. Labeling Strategy for STED Nanoscopy

As STED nanoscopy continues to advance towards achieving nanometric resolution, the effective resolution attainable is significantly impacted by the labeling approach utilized to attach the fluorescent probe to the protein of interest. In immunocytochemistry, the common practice involves utilizing primary antibodies coupled with multiple-dye-labelled secondary antibodies, which enhances molecular specificity and signal-to-noise ratio. However, a notable drawback to this method is the relatively large position uncertainty in proxying the target molecule, which can range between 15 and 20 nm, falling within the resolution range of STED nanoscopy. Consequently, developing other effective labeling strategies with high spatial precision becomes relevant.

### 5.1. Single-Domain Antibodies and Affibodies

As alternative to using antibodies as labeled targeting molecules, different classes of smaller affinity labels have been developed, which, due to their smaller size, offer higher spatial precision in labeling.

Single-domain antibodies (sdAbs), also known as nanobodies, comprise a single monomeric variable domain of the heavy chain, and have molecular weights of approximately 12–15 kDa and small dimensions of around 3 nm. Compared to traditional antibodies, nanobodies exhibit higher solubility, efficient expression and, most importantly, a small size that minimizes position uncertainty when used for immunocytochemistry [38,39,40]. Therefore, the use of nanobodies is increasingly being considered as a labeling strategy for STED nanoscopy [24,91,92]. Affibodies, which are small, engineered proteins based on a three-helix bundle domain, have previously been used in the context of specific molecular targeting in diagnostic and therapeutic applications [93,94]. Until now, there have been a limited number of reports using affibodies for STED imaging [95,96]. Nevertheless, their small size (about 6 kDa) makes them promising labels as a means to promote spatial precision in super-resolution optical microscopy.

However, the dearth of nanobodies and affibodies targeting native proteins remains a challenge, with only a small number of high-affinity nanobodies or affibodies with native targets being commercially available, and even fewer that have undergone rigorous testing and are freely accessible to the scientific community. To overcome this challenge, it may be possible to expand their availability by targeting frequently utilized fusion tags, such as GFP [38]. Therefore, while nanobodies and affibodies hold promise as probes for STED nanoscopy, further development and expansion of their availability will be necessary for widespread applications.

### 5.2. Genetically Encoded Protein Tags

Genetically encoded protein tags, including fluorescent proteins, as well as small tags that enable bio-orthogonal labeling with organic fluorophores, have emerged as promising labeling strategies in STED nanoscopy [97,98,99,100]. Fluorescent proteins provide superior positional accuracy relative to antibody pairs, as well as significant convenience in that they possess inherent fluorescence and obviate the need for additional fluorescent probes in labeling applications. Nevertheless, they frequently exhibit low photostability and brightness, thus limiting their utility in certain contexts. Fluorescent proteins suitable for utilization in STED nanoscopy have been developed with the aim of achieving increased photostability and enhanced fluorescence brightness [41,42,43,44,45,46,47,48,49,50]. Small tags (~30 kDa or smaller) that facilitates bio-orthogonal labeling, such as Snap-tags or Halo-tags, can also enable high positional accuracy in labeling while simultaneously leveraging an extensive repertoire of pre-existing organic fluorophores labelled to them [51,52,53].

### 5.3. Genetic Code Expansion

A recent advancement in labeling strategies involves genetic code expansion through the incorporation of unnatural amino acids (or non-canonical amino acids, ncAAs) that can be labeled via ‘click’ chemistry. This approach permits the site-specific labeling of target proteins by directly attaching a fluorophore to the protein, thereby enabling direct reporting of the protein’s location [54,55]. Although this method is relatively new and poses some technical challenges, it exhibits significant potential for nanoscopic imaging tasks that necessitate exceptional labeling positional accuracy, such as in advanced STED nanoscopy, and an even higher potential for SRM modalities pushing the spatial resolution into the nanometer range and beyond.

## 6. Discussion

**Figure 7:**
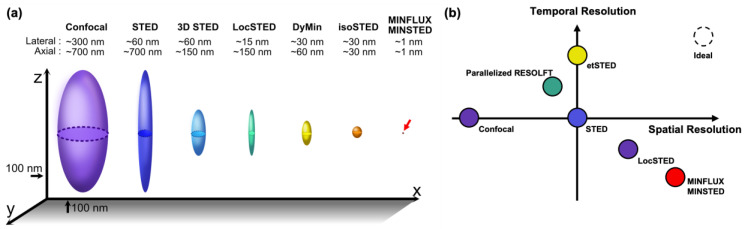
(**a**) Overview of 3D spatial resolutions and (**b**) brief perspective diagrams of spatiotemporal resolutions of selected recently developed STED nanoscopy methods.

STED optical nanoscopy has emerged as a revolutionary technique in the field of life sciences, surpassing the resolution limits of conventional far-field optical microscopy, while retaining its advantages, such as non-invasiveness and live cell imaging. Nevertheless, the trade-off relationship between various parameters, such as the spatial resolution, temporal resolution, field-of-view (FOV), phototoxicity, and imaging depth, that exists in optical microscopy, also applies to STED nanoscopy. In this review, we have presented some strategies that, in different ways, can provide remedies to these trade-off relationships, and which provide means to push the limits of the spatial and temporal resolution of STED nanoscopy. With recent advances combining STED and SMLM principles, such as in MINFLUX and MINSTED, the spatial resolution of STED nanoscopy has reached the nanometer or sub-nanometer scale [21,22,66,67,68,69]. Other techniques, such as 3D STED and isoSTED, have significantly enhanced the axial resolution of STED nanoscopy [10,13,33,34,35]. To achieve improved temporal resolution, clever strategies, such as parallelized illumination or event-based triggering, have been employed [19,36,37,88,89,90]. In Figure 7, we plotted some of the notable recent developments in STED nanoscopy to provide a perspective in terms of their spatiotemporal resolution. The lateral and axial resolution of the methods are illustrated as 3D PSFs in Figure 7a. The trade-off between the spatial and temporal resolution of each method is depicted in Figure 7b. It is important to note that the resolution achieved in any fluorescence microscopy is contingent upon the location accuracy of the fluorescent probe used to label the molecule of interest. Therefore, we also discuss recent efforts to develop labeling strategies that offer enhanced position accuracy down to the nanometer scale [38,39,40,41,42,43,44,45,46,47,48,49,50,51,52,53,54,55].

The development of STED nanoscopy has seen significant strides made, but there are still opportunities for further advancement. Achieving resolution enhancement without photo switching remains limited to enhancements to the order of tens of nanometers. At the same time, the temporal resolution of STED nanoscopy generally falls short compared to many other lower-resolution techniques, such as structured illumination microscopy. Furthermore, to improve compatibility with live cell imaging, it is important to further address the issue of phototoxicity. While not discussed in the main text, the maintenance of high resolution when imaging thicker samples at greater depths is also crucial in advancing the capabilities of STED nanoscopy. To achieve this goal, it is imperative that continued improvements can be made in this direction, which may involve the further extension of STED imaging into the NIR, or the implementation of adaptive optics techniques or other novel approaches [101,102]. As one of several modalities for super-resolution imaging, with their pros and cons, the pursuit of improved resolution and imaging capabilities in STED nanoscopy will undoubtedly continue to drive research and development efforts in the future.

## Data Availability

No new data were created or analyzed in this study. Data sharing is not applicable to this article.
